# Comparative analysis of sorghum (C4) and rice (C3) plant headspace volatiles induced by artificial herbivory

**DOI:** 10.1080/15592324.2023.2243064

**Published:** 2023-08-10

**Authors:** Cyprian Osinde, Islam S. Sobhy, David Wari, Son Truong Dinh, Yuko Hojo, Dandy A. Osibe, Tomonori Shinya, Arthur K. Tugume, Anthony M. Nsubuga, Ivan Galis

**Affiliations:** a Department of Plant Sciences, Microbiology and Biotechnology Makerere University, Kampala, Uganda; b Institute of Plant Science and Resources, Okayama University, Kurashiki, Japan; c Department of Plant Protection, Faculty of Agriculture, Suez Canal University, Ismailia, Egypt; d School of Biosciences, Cardiff University, Cardiff, UK; e Faculty of Biotechnology, Vietnam National University of Agriculture, Hanoi, Vietnam; f Department of Plant Science and Biotechnology, University of Nigeria, Nsukka, Nigeria

**Keywords:** Volatile organic compounds, rice, sorghum, indirect defense, herbivores, terpenoids

## Abstract

Acute stress responses include release of defensive volatiles from herbivore-attacked plants. Here we used two closely related monocot species, rice as a representative C3 plant, and sorghum as a representative C4 plant, and compared their basal and stress-induced headspace volatile organic compounds (VOCs). Although both plants emitted similar types of constitutive and induced VOCs, in agreement with the close phylogenetic relationship of the species, several mono- and sesquiterpenes have been significantly less abundant in headspace of sorghum relative to rice. Furthermore, in spite of generally lower VOC levels, some compounds, such as the green leaf volatile (*Z*)-3-hexenyl acetate and homoterpene DMNT, remained relatively high in the sorghum headspace, suggesting that a separate mechanism for dispersal of these compounds may have evolved in this plant. Finally, a variable amount of several VOCs among three sorghum cultivars of different geographical origins suggested that release of VOCs could be used as a valuable resource for the increase of sorghum resistance against herbivores.

## ONE SENTENCE SUMMARY

This paper shows how genetically related plants with similar volatile toolboxes define their own species identity in the ecological space.

## Introduction

During coevolution with insects, plants developed a complex arsenal of defense mechanisms against antagonistic herbivores.^1,2^ One of these defenses is synthesizing and releasing a complex bouquet of volatile organic compounds (hereafter called VOCs) in response to insect attack.^3,4^ An important subset of plant VOCs is induced by herbivory and therefore termed as herbivory-induced plant volatiles (HIPVs). The VOCs, and HIPVs in particular, play exceedingly important roles as infochemicals in the interactions between plants and insects.^5^ Whereas herbivorous insects make use of plant volatiles to locate host plants as food,^6^ the success of natural enemies in locating their host (parasitoids) or prey (predators) also depends on these signals.^7^

While details on biosynthesis and release of VOCs have already been investigated in multiple dicots,^8–10^ relatively less is known about the volatile emissions from monocot plant species.^11^ In addition, the efficacy and potential differences in volatile emissions from C3 and C4 monocotyledonous plants have not yet been directly addressed. The best studied individual monocot models to date include maize (C4 plant; *Zea mays* L.),^12^ and rice (C3 plant; *Oryza sativa* L.);^13^ however, additional studies are needed to expand the knowledge of monocot VOCs. For example, sorghum (C4 plant; *Sorghum bicolor* L.) is the fifth most important cereal, which is, however, quite susceptible to herbivores in the field.^14,15^ In our previous reports, sorghum suffered up to 6% total leaf damage in the field,^16^ while Nipponbare model rice had only about 1% of total leaf damage.^17^ Such observations put in question the relative effectiveness of sorghum defense barriers against insect herbivores, which may, in part, be due to limited and/or impaired release of defense-related VOCs from sorghum plants. In particular, as volatile bouquets of sorghum and rice are reportedly quite similar,^13,18^ the amounts at which the volatiles are released to headspace need to be addressed. Direct comparative studies using sorghum and rice are therefore useful to gain more information, and possibly design novel methods for the improvement of sorghum resistance against herbivores.

In the initial working hypothesis, we proposed that differential life strategies and core metabolic systems (C3 vs. C4) might be modulating individual VOCs in rice and sorghum, which in turn, is affecting the intensity of indirect defenses, and causes differential damage of plants in the field. In order to test the first installment of this hypothesis, volatile entrainment was conducted before and after the plants were treated with artificial infestation of generalist herbivore, Loreyi armyworm (*Mythimna loreyi* Duponchel), and the collected VOCs of sorghum and rice, respectively, were analyzed by GC-MS. Our results provide evidence that both plant species displayed a surprisingly large quantitative differences in headspace VOC composition, particularly mono- and sesquiterpenes, which were generally lower in sorghum compared to rice. Further, to explore the underpinning molecular mechanism involved in the VOC emissions in three sorghum varieties, we examined the expression of a set of sorghum volatile genes involved in terpene biosynthesis. Our findings open additional avenues for exploiting the defense traits of monocot plants against insect herbivores,^19^ as well as open questions about the possible differences among various C3 and C4 plant species in the emissions of VOCs.

## Materials and methods

### Plant materials

Seeds of sorghum (*Sorghum bicolor* (L.) Moench) varieties (BTx623, NOG, and Epuripur) were germinated in Petri dishes on wet filter paper for one week, and then transferred to 100 cc plastic pots with moistened Tanemaki baido gardening substrate (Takii ®, Kyoto, Japan). Three sorghum cultivars used in the study are adapted to distinct geographic regions: BTx623 is a standard American inbred line;^20^ NOG is a landrace from Japan;^21^ and Epuripur is a recently selected cultivar for beer brewing in Uganda (Africa).^22^ Rice (*Oryza sativa* L.) seeds of *japonica* variety Nipponbare were germinated in nutrient-rich soil pellets Kumiai Ube Baido No.2 (MC Ferticom, Tokyo, Japan) in 7 × 7-well (L1.5 × W1.5 × D2.5 cm) trays (L15 × W15 cm) standing in water maintained at approximately 1–2 cm depth level. Two weeks later, germinated plantlets were transferred to individual 100 cc pots with sterilized field soil mixed with Ube Baido in a 4:1 (v/v) ratio. Plants were maintained in the cultivation room at 14 h photoperiod, temperature 28 ± 3°C, and outdoor (window) irradiation supplemented with generic fluorescent lights (total light intensity 80–120 µmol m^−2^ s^−1^). Sorghum plants were watered every second day to keep soil moistened while rice pots were continuously maintained in plastic trays (L50 × W35 × D6 cm) filled with water at 4–5 cm depth level. Rice and sorghum plants at 4 to 6-week age were used for artificial herbivory treatments, VOC collections, and RNA extractions.

### Treatment mimics of the attack by M. loreyi armyworm larvae

Oral secretion (OS) from Loreyi armyworm *Mythimna loreyi* Duponchel (Lepidoptera: Noctuidae) was used for plant elicitation as described for other insects in Alamgir et al.^23^ Typically, larvae were kept in the laboratory on a pinto bean-based artificial diet after neonates emerged from eggs laid on rice in the insect cage. The OS was collected from 4–5^th^ larval instars that were feeding on rice leaves for 2–3 days prior to OS collection. Each larva was immobilized between fingertips and aggravated with a blunted micropipette tip to induce defensive regurgitation. Regurgitate (OS) was collected using a vacuum device as described in Shinya et al.^24^ Concentrated OS was stored in closed plastic tubes at − 80°C. A layer of nitrogen gas was applied over the liquid surface before closing. Prior to experiments, OS aliquots were thawed on ice, briefly centrifuged at maximum speed and supernatants were diluted 3-fold with deionized water before application on wounded leaves to mimic the plant responses induced by real *M. loreyi* attack (referred to as “artificial herbivory”). At 3PM, two youngest fully developed leaves of sorghum varieties (BTx623, NOG and Epuripur) either at the 4- or 6-week stage, and two youngest fully developed leaves of 6-week-old rice (Nipponbare) were wounded with a fabric pattern wheel along the midvein, and fresh wounds were immediately treated with 15 µL diluted OS per leaf. VOCs were collected immediately after artificial herbivory treatment for 24 hours, except for diurnal experiments. In diurnal volatile collections, artificial herbivory was conducted in two steps, first by treating basal leaf parts with 15 µL diluted OS at 3PM, which was followed by upper leaf part treatments at 5PM. Applying oral secretion in two space- and time-divided intervals was employed to simulate repeated herbivore attacks.^25,26^ VOCs were collected in 3-hour time intervals, starting in the next day morning at 6AM, and continued for a 27-hour day/night period. The overnight rest period after treatments was used to allow partial healing of wounds and thus collection of true diurnally-regulated VOCs, devoid of passively escaped volatiles *via* freshly open wounds.

### Volatile collections

Trapping of headspace VOCs was carried out essentially as described in Sobhy et al.^27^ Pots with treated and untreated plants were inserted into a small Ziplock bag and sealed around stems to reduce the levels of nonspecific soil volatiles. Rice plants were supplied with 30 mL of water in each bag, while sorghum in wet soil was used without water. Two plants for each treatment were carefully inserted into an acrylic cylinder (H50 × i.d.15 cm) with a sealed top. Two ports in each cylinder were used for air circulation and VOC collection: the lateral port 15 cm from the base was used as an air inlet, and the second port on the top served as an air outlet. The inlet air partially purified with charcoal filter was pulled into cylinders at approximately 0.75 L min^−1^ by suction force applied to outlet ports. Each outlet port was equipped with a custom-made 10 cm glass trap (5 mm i.d.) packed with Porapak Q sorbent (200 mg, Supelco Analytical, Bellefonte, USA). Sorbent was held in place by two plugs of deactivated glass wool (Shimadzu, Japan). All collection cylinders (*n* = 12) were connected to single ULVAC DAP-12S 167 vacuum pump (ULVAC KIKO. Inc., Japan) adjusted to generate air flow of 10–15 L min^−1^. Before trapping, the basal part of each collection cylinder was submerged in water to seal the whole system. VOCs were collected for 24 h using a single trap (total volatiles), or traps were replaced every 3 h for the collection of diurnal volatiles at specified times, resulting in a single or nine collection sample sets, respectively.

### Analysis of volatiles

Collected volatiles were eluted with 1 mL dichloromethane (DCM) after 400 ng tetralin (1,2,3,4-tetrahydronaphthalene, Nacalai Tesque, Japan) internal standard dissolved in 5 μL DCM was applied to Porapak Q filter traps by glass microsyringe (Hamilton Company, Inc., Reno, USA). Samples in 1.5 mL glass vials with PTFE liner caps (Supelco Analytical, USA) were analyzed on Agilent 7891A GC/Agilent 240 MS equipped with a HP-5 MS column (5% Phenyl Methyl Silox, 30 m length x 0.25 mm inner diameter x 0.25 µm film thickness, Agilent Technologies, USA). One µL of Porapak Q eluate in DCM was introduced to GC in split mode (3:1) by autosampler (Agilent 7693A) *via* injector port held at 230°C. Helium was used as carrier gas at a flow rate of 1 mL min^−1^. The ion trap was held at 200°C, transfer line was 260°C, and emission current was 30 µAmps. GC oven was held at 40°C for 3 min, and then the temperature was increased by 5°C min^−1^ to 180°C, followed by 20°C min^−1^ ramp to 300°C, and 5 min holding time. MS data were collected in full scan mode narrowed to *m/z* 40–300 mass range. Peaks in chromatograms were analyzed by Agilent Workstation 7.0.2. Quantification of known compounds with standards was performed by comparison of peak areas to external authentic standards applied to GC-MS in concentration range of 0.1–5 ng/μL. For compounds without authentic standards, quantification was performed relative to a near class compound, such as linalool for monoterpene calibrations, and β-caryophyllene for sesquiterpenes.

### Gene expression

Transcript levels of sorghum genes were determined by quantitative RT-PCR as described in Shinya et al.^24^ Total RNA was extracted from approximately 100 mg tissue by Trizol (Invitrogen, USA) and cDNA was synthesized with PrimeScript reverse transcriptase (Takara Bio Inc., Japan) after DNase treatment and purification of total RNA. Transcript levels were determined by THUNDERBIRD qPCR Mix on a CFX Connect^TM^ Real-Time System (Bio-Rad Laboratories, Inc., USA). The *SbEF1α* (Sb10g023360) housekeeping gene was used to normalize the relative transcript levels in sorghum based on our previous experience and use of *OsEF1α* (Os03g0177900) for normalization of rice genes.^13^ Gene-specific oligonucleotide primers for qRT-PCR are summarized in Table S3.

### Statistical analysis

PCA analysis and clustering were conducted with default parameters in MetaboAnalyst ver. 5.0 tool (https://www.metaboanalyst.ca/).^28^ ANOVA test of variance followed by Tukey’s HSD was performed with *multcompView* package in R.^29^ The Student’s *t*-test was performed with Microsoft Excel. Prior to statistical analyses, normality of data was examined by Shapiro-Wilk test in OpenStat (http://statpages.info/miller/OpenStatMain.htm), and if data showed evidence against normality, values were *log* transformed before statistical tests.

## Results

### Profiling of sorghum and rice volatiles

**Figure 1.**
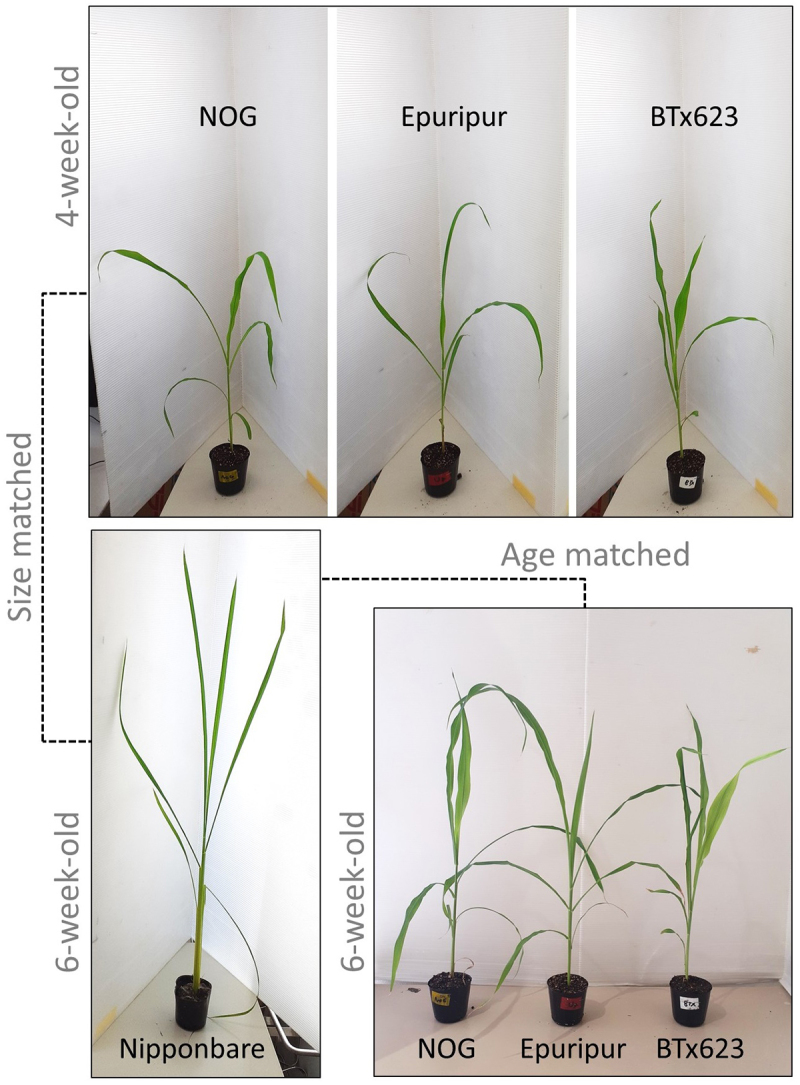
Visual comparison of sorghum and rice plants used in experiments.

**Figure 2.**
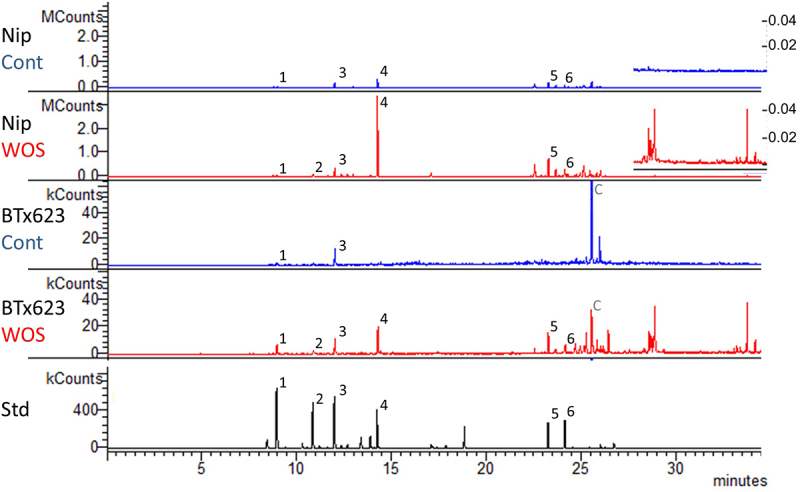
Comparison of sorghum and rice volatile profiles.

In the laboratory, sorghum and rice show differential growth rates, which makes direct comparisons of headspace VOCs complicated. To address this issue, we compared standard rice cultivar Nipponbare and three sorghum varieties (BTx623, Epuripur, and NOG) when the plants attained (1) similar size, i.e., 6 week-old rice and 4 week-old sorghum plants, and when (2) plants reached the same age, i.e., when both rice and sorghums were 6-week old (Figure 1). At first, we focused on the VOC profiles released from the size-matched rice and BTx623, using untreated (control) and artificial herbivory-treated (WOS) plants. Both sorghum and rice emitted a qualitatively similar bouquet of VOCs, however, at the same time, it was noticeable that both species displayed large quantitative differences, despite the emitting plants were of similar size. While many rice terpenoids, represented by extracted ion trace *m/z* 93, could be easily found in headspace of rice, majority of sorghum peaks were much less intense (Figure 2). Based on the authentic standard co-injections, the monoterpenes α-pinene, myrcene, D-limonene and linalool, and the sesquiterpenes β-caryophyllene and β-farnesene were identified at expected retention times in headspace samples from rice and sorghum. In addition, a number of other less abundant tentatively-identified compounds was common in rice and sorghum, as revealed by the Agilent Workstation 7.0.2. peak integration (Table S1).

Sorghum and rice plants were potted in soil and grown in cultivation room for 4 and 6 weeks (sorghum) or 6 weeks (rice). Upper panel shows that size of sorghum plants at 4 weeks is comparable to rice at 6 weeks (lower panel, left). Sorghum at 6 weeks produces broader leaves and becomes larger than rice at the same age.

The ion fragment *m/z* 93 representing terpenoid compounds was extracted from chromatograms of untreated rice (Nip-Cont), artificial herbivory-treated rice (Nip-WOS), untreated sorghum (BTx623-Cont) and treated sorghum (BTx623-WOS). Bottom chromatogram shows mixture of standard compounds (Std) run under the same conditions in GC-MS. In order to show peaks in sorghum, chromatograms are magnified 50× (scale 0-50 kCounts) compared to rice (scale 0-2.5 MCounts). Representative compounds occurring in both plants are labeled with numbers: 1, α-pinene; 2, myrcene; 3, D-limonene; 4, linalool; 5, β-caryophyllene; 6, β-farnesene; C, contaminant peak of butylated hydroxytoluene.

### Herbivory-induced volatile emissions

**Figure 3.**
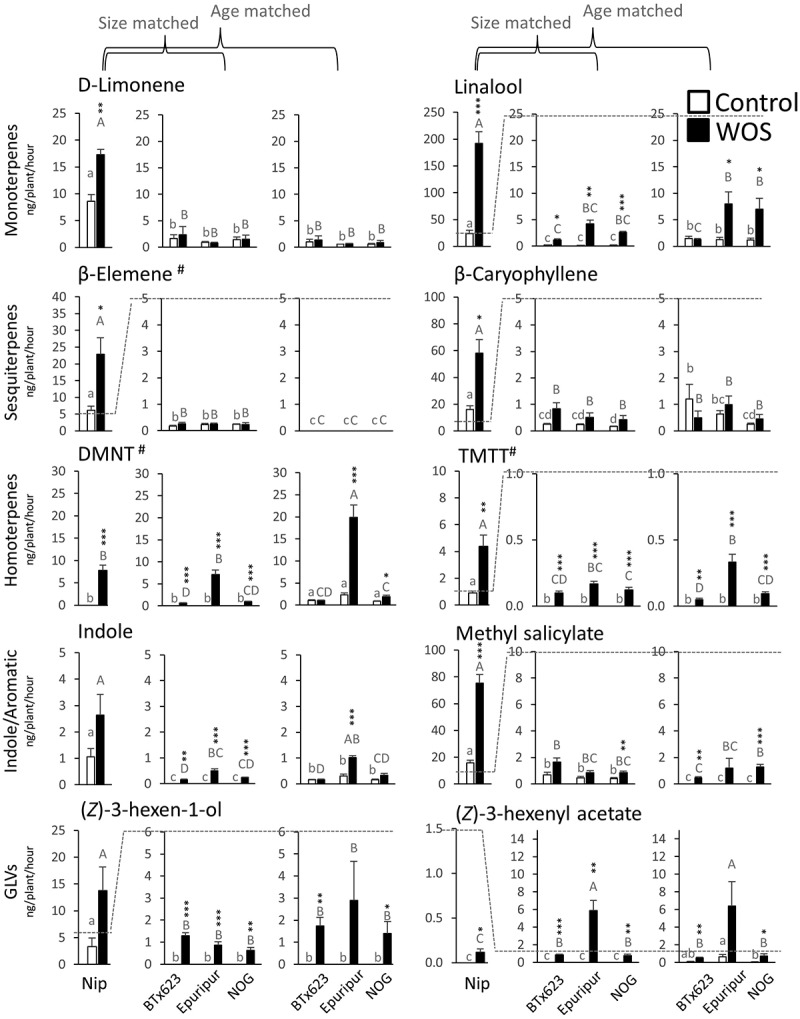
Accumulation of major VOCs in headspace of sorghum and rice.

The main HIPVs in rice were identified as monoterpene linalool; sesquiterpenes β-elemene and (*E*)-nerolidol; homoterpenes (*E*)-4,8-dimethyl-1,3,7-non-atriene (DMNT) and (*E,E*)- 4,8,12-trimethyl-1,3,7,11-tridecatetraene (TMTT); GLVs (*Z*)-3-hexen-1-ol and (*Z*)-3-hexenyl acetate; and aromatic compounds indole and methyl salicylate^13^ (Figure 3, Table S1). All these HIPVs, except for indole and (*Z*)-3-hexen-1-ol, were significantly more abundant in headspace of rice plants treated with artificial herbivory (Figure 3, Table S1). Upon the same treatment, a similar set of HIPVs was induced in sorghum, supporting the overall VOC conservation in sorghum and rice. However, as noticed above, strong quantitative differences could be detected in both species (Figure 3, S1, Table S1). Sorghum headspace contained approximately 1/10 of linalool, TMTT, methyl salicylate and (*Z*)-3-hexen-1-ol, while it showed a somewhat more comparable level of indole and DMNT with rice. In contrast to generally low terpenes found in sorghum headspace, (*Z*)-3-hexenyl acetate was more abundant in sorghum relative to rice. The differences have been preserved in 6-week-old sorghum (Figure 3), suggesting that even older sorghum plants cannot produce more volatiles, despite their size is bigger than that of rice (Figure 1).

Three sorghum varieties (BTx623, Epuripur and NOG), either size or age matched with rice plants, were placed in acrylic cylinders and VOCs were captured by Porapak filters. After elution and GC-MS runs, compound peaks in specific ion chromatogram traces were quantified and the content of monoterpenes, sesquiterpenes, homoterpenes, indole/aromatic compounds, and green leaf volatiles (GLVs) was determined against external calibration curves. For compounds without authentic standards (labeled with ‘#’), the content was quantified against representative compounds from a similar structural group (DMNT as linalool equivalent; β-elemene and TMTT as β-caryophyllene eq.). Data are means from independent samples (*n = 4*) with SE. Asterisks show significant differences between each pair of control (untreated) and WOS-treated plants (*P ≤ 0.05, **P ≤ 0.01, ***P ≤ 0.001). Different small letters show significant differences for each compound within the control group of samples (Control) determined by ANOVA with Tukey LSD. Differences within the treated group of samples (WOS) for each compound are shown with different capital letters.

**Figure 4.**
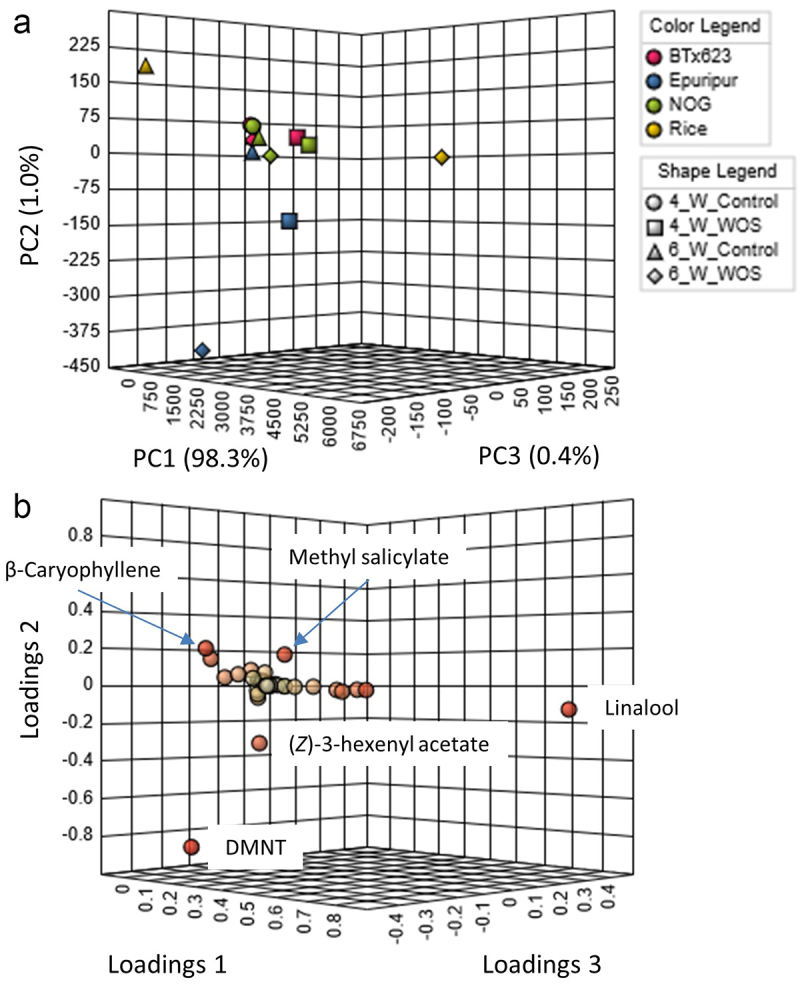
Principal component analysis (PCA) of VOCs in headspace of sorghum and rice.

In accord with quantitative differences observed in VOCs of rice and sorghum (Figure 3), principal component analysis (PCA) showed a clear separation of rice from all sorghum cultivars (Figure 4a), both at control and induced levels. Out of 39 compounds used in PCA analysis, major separations between rice and sorghum appeared in linalool, DMNT, (*Z*)-3-hexenyl acetate, methyl salicylate, and β-caryophyllene (Figure 4b). Interestingly, one of the sorghum cultivars, Epuripur, also separated in PCA plot from the two other genotypes, thus revealing a significant level of genetic variation in the production and/or release of VOCs in sorghum. Apparently, Epuripur headspace contained relatively more HIPVs compared to other sorghums, which mainly involved the important HIPVs linalool, DMNT and (*Z*)-3-hexenyl acetate (Figure 3).

VOCs in sorghum and rice headspace shown in Figure 3 and Table S1 were subjected to PCA analysis using MetaboAnalyst tool. (a) 3D-PCA plot shows a clear separation of rice VOCs (yellow) from sorghum samples (BTx623, red; Epuripur, blue; NOG, green). A separation also occurs between Epuripur and two other sorghums. Circles represent untreated 4-week-old plants (control); squares are 4-week-old plants treated with artificial herbivory (WOS); triangles are untreated plants at 6 weeks; diamonds are 6-week-old plants treated with WOS. (b) Loading plots show compounds with the highest contribution to the separation of headspace volatiles in rice and sorghum.

### Diurnal volatile emissions

**Figure 5.**
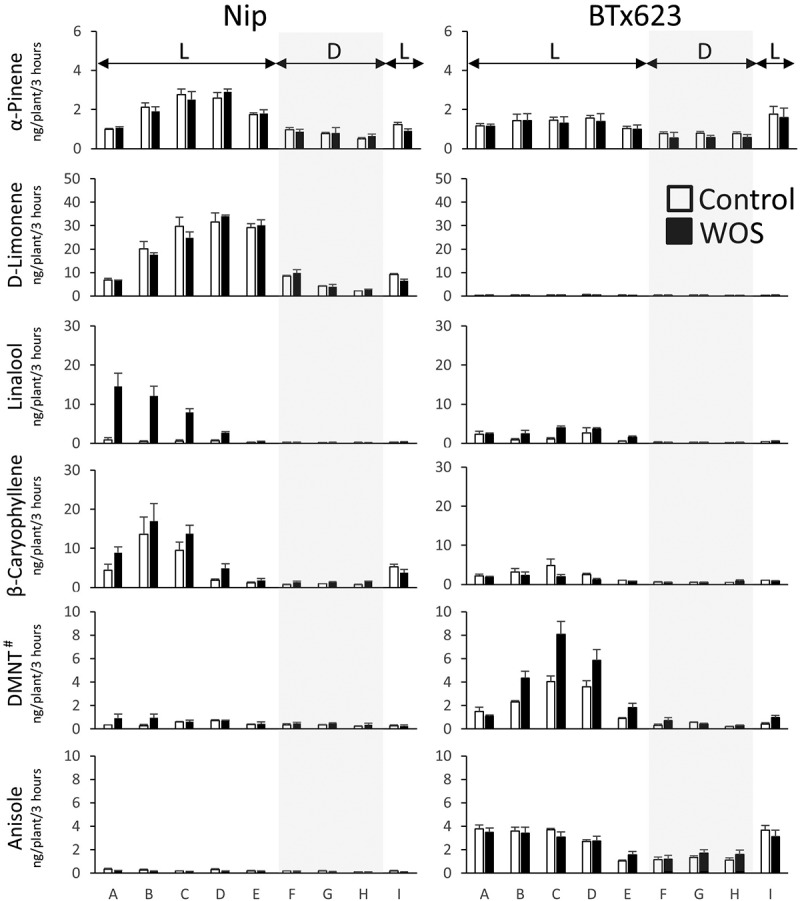
Diurnal profiles of headspace VOCs released from C3 rice (Nipponbare) and C4 sorghum (BTx623).

Next, we asked if daytime emissions are responsible for differential amounts of VOCs observed in initial bulk 24 h entrapments (Figure 3). We thus conducted a time-resolved collection of VOCs under control and artificial herbivory conditions. In this experiment, however, plants were allowed to rest one night after treatment to allow partial healing of open wounds inflicted by artificial herbivory. This was used to obtain more specific diurnal patterns, i.e. those which are devoid of VOCs that passively escape from the plant’s open wounds (see Materials and Methods). In result, both sorghum and rice volatile emissions followed diurnal rhythm (Figures 5, S2), although total number of volatiles detectable in the short 3 h trapping periods was reduced, due to volatiles falling below the detection limit of GC-MS instrument (Table S2). However, majority of previously observed trends and differences in volatiles between sorghum and rice were conserved. For instance, Epuripur plants released comparably more HIPVs (e.g. DMNT and (*Z*)-3-hexenyl acetate) than BTx623 and NOG (Figures 5, S2, Table S2). Anisole release was predominant in BTx623, which was associated with the photoperiod (Figures 5, S2).

Sorghum and rice plants at 6 weeks were placed in acrylic cylinders and VOCs were trapped on Porapak filters in 3 h intervals (A, 6AM-9AM; B, 9AM-12AM, C, 12AM-3PM; D, 3PM-6PM; E, 6PM-9PM; F, 9PM-12PM; G, 12PM-3AM; H, 3AM-6AM; I, 6AM-9AM). Plants were either untreated (Control) or treated with artificial herbivory (WOS) twice on the previous day at 3PM and 5PM. Data are means from independent samples (*n = 3*) with SE. L, light period, D, dark period.

### Expression of sorghum terpene synthases

**Figure 6.**
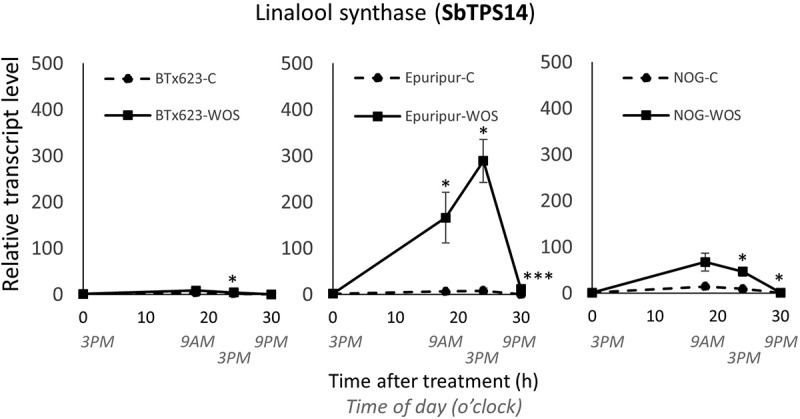
Expression analysis of putative sorghum linalool synthase SbTPS14.

Notes: Sorghum cDNAs were prepared from control and WOS-treated sorghum samples. RT-qPCR was performed to determine transcript abundances of sorghum linalool synthase in BTx623, Epuripur and NOG leaves. Data are means ±SE (*n = 3-4*). Asterisks show significant differences determined by Student’s *t*-test between pairs of control (untreated) and WOS-treated plants at the same time point (*P ≤ 0.05, ***P ≤ 0.001).

As VOCs appeared quantitatively different in three sorghum cultivars, we examined the cultivar-specific expression of terpene synthases.^18^ The transcript levels of *SbTPS3* (Sb07g004480), *SbTPS4* (Sb07g005130), *SbTPS5* (Sb07g003080), *SbTPS14* (Sb04g001780), and a putative sorghum *hydroperoxide lyase* (*SbHPL*, Sb04g000830), were determined in control and artificial herbivory-treated BTx623, Epuripur and NOG plants. *SbTPS3* gene transcripts were strongly induced in NOG and Epuripur (Figure S3A), supporting the inducible character of the main TPS3 enzymatic products, (*E*)-β-farnesene and (*E*)-α-bergamotene. *SbTPS4* gene expression was suppressed by artificial herbivory (Figure S3B), same as caryophyllene synthase in rice.^13^ *SbTPS4* expression was not particularly correlated with the volatile β-caryophyllene levels shown in Figure 3. The *SbTPS5* showed an inconsistent expression pattern in sorghum (Figure S3C). Interestingly, transcripts of *SbTPS14*, here assigned as putative sorghum linalool synthase (Figure S4), were strongly elevated by artificial herbivory. Furthermore, *SbTPS14* transcripts were more abundant in Epuripur relative to NOG and BTx623 (Figure 6), which was consistent with the observed headspace linalool levels (Figure 3). Putative sorghum *SbHPL* gene, identified based on the protein similarity with rice SbHPL3 (Figure S5), was downregulated in the morning (9AM), and, generally, transcripts were not changed by artificial herbivory (Figure S3D), similar to previous *OsHPL3* report in rice.^13^

## Discussion

In this study, sorghum and rice released similar VOCs, as might have been expected from a close phylogenetic relationship of the species.^30–32^ However, in direct comparison, sorghum and rice displayed large quantitative differences in their headspace VOC contents. In particular, levels of several mono- and sesquiterpenes were dramatically lower in sorghum compared to rice. On the other hand, homoterpene DMNT showed more similar contents in rice and sorghum, and sorghum had greater GLV (*Z*)-3-hexenyl acetate emissions than rice. In addition, three sorghum cultivars had somewhat different volatile profiles, showing that intra-species genetic variation exists in sorghum for the production and/or release of VOCs.

The spectrum of sorghum VOCs identified in this study was consistent with previous independent study by Zhuang et al.,^18^ in which the fall armyworm (*Spodoptera frugiperda*) feeding induced headspace levels of monoterpene linalool, sesquiterpenes β-elemene, (*E*)-β-caryophyllene, (*E*)-α-bergamotene, sesquisabinene A, (*E*)-β-farnesene, α-humulene, zingiberene, β-bisabolene, β-sesquiphellandrene and (*E*)-nerolidol, GLV (*Z*)-3-hexenyl acetate, and aromatic compound, indole. In our experiments (Table S1), although we did not find sesquisabinene A and zingiberene, we additionally detected DMNT and TMTT, two homoterpenes commonly present in monocot plants,^33^ and another aromatic compound methyl salicylate, in the sorghum headspace. Anisole was identified as volatile predominantly released by BTx623 sorghum, relative to rice and two other sorghum cultivars (Figure 5, S2). Similar to rice, most of the sorghum VOCs were emitted during daytime (Figure 5, S2).

In spite of qualitative overlap between volatile blends of rice and sorghum (Table S1, Figures 3, 5, S2),^13,34,35^ significantly higher levels of induced VOCs were typical for rice. In addition, low levels of basal volatiles emitted from sorghum support the idea of sorghum being a low emitter rather than being a low responder to herbivory stimuli. In the natural environment, sorghum and rice have evolved very distinct lifestyles. Sorghum, which is a C4 plant, is more efficient in carbon-fixation, water and nitrogen utilization, and it performs well under extreme temperatures and light.^36,37^ In contrast, a representative of C3 plants, rice, is highly dependent on water and nutrient supply to achieve the expected yield.^38^ With respect to quantitative VOC differences between rice and sorghum, it is possible to infer from a large volume of indirect evidence that C3 and C4 metabolic networks might be actually involved in the empirically observed differential emission of VOCs in our experiments. Previously, Niinemets and Reichstein^39,40^ concluded that the amounts of VOCs released by plants are affected by stomatal conductance, which, reportedly, tends to be lower in C4 compared to C3 plants, especially under high light conditions.^41–43^ In 28 grass species, C4 species consistently had lower maximum stomatal conductance to water (g_*max*_) than their C3 relatives.^44^ Therefore, C3 and C4 plant adaptations may be helpful to explain the differential release of VOCs, clearly exemplified here by the rice and sorghum headspace analyses.

However, alternative explanations for differential VOCs in rice and sorghum should also be considered. For example, sorghum is a typical high biomass crop, which may divert all resources to growth on the account of defense. This hypothesis is supported by observations of plant behaviors that involve trade-offs between growth and defense.^45,46^ Another possibility is that sorghum preferentially retains volatiles inside for the purposes of direct defense.^47^ Specific plants are known to retain large amounts of terpenoids, such as mint *Teucrium marum* storing monoterpenes in the leaf epidermal capsules.^48^ In our unpublished metabolomics experiment, we noticed that several annotated metabolites in sorghum correspond to volatile conjugates. However, further investigations are needed to confirm (1) the identity of these compounds (2) and their role as potential internal stores of terpenoids in sorghum. Furthermore, while silicon in rice has been reported to promote VOC release,^49^ waxy cuticles in sorghum might be preventing the passive diffusion of volatiles in headspace.^50^ Finally, volatiles in sorghum could be functionally tuned down for a lower activity, possibly due to “redundancy” in defense implied from the evolution and use of the potent cyanogenic glycoside dhurrin in sorghum species.^51^

Genetic diversity in sorghum is well studied, however, most reports remain focused on growth, yield, and nutritional quality of plants.^52,53^ Three sorghum cultivars in this study showed quantitative metabolic differences, namely in linalool, DMNT, and (*Z*)-3-hexenyl acetate (Figures 3, 4B), which contents were higher in Epuripur headspace (Figure 3). Previously, (*Z*)-3-hexen-1-ol acetate was dominant (65%) in volatile blend of 4-week-old sorghum,^54^ and this compound was important for attractiveness to shoot fly (*Atherigona soccata*) in GC-EAG.^55^ In the same study, volatile emissions from two sorghum cultivars, Swarna and IS 18551, were compared, and, interestingly, IS 18551 cultivar, which is resistant to *A. soccata*, emitted less VOCs compared to susceptible Swarna. In other plants, tea green leafhopper (*Empoasca vitis*) selected host plants based on emissions of (*Z*)-3-hexenyl acetate.^56^ Therefore, differential emissions of volatiles from the closely related varieties, while being of practical importance, also open theoretical questions on the regulatory mechanisms that underpin these differences.^57–59^ As sorghum genome and TPS genes have already been known,^18,31,60^ *SbTSP3*, *SbTSP4*, *SbTSP5*, *SbTSP14*, and *SbHPL* gene expression was examined in this study. *SbTPS3* and *SbTPS14* were strongly induced by artificial herbivory (Figures 6, S3). Furthermore, *SbTPS14* transcript levels were strongly induced and consistent with the observed headspace linalool levels in three sorghum cultivars (Figure 3): BTx623 (lowest), NOG (intermediate) and Epuripur (highest). In contrast, transcript levels of *SbHPL* for GLV production, including (*Z*)-3-hexenyl acetate, were not regulated by artificial herbivory, and transcripts fluctuated diurnally (Figure S3D). These data suggest that while in some cases the release of volatiles could be dependent on the variety-specific expression of biosynthetic genes (e.g., linalool), in other cases, such as GLVs, volatiles may be more dependent on the substrate availability, or other control mechanisms in VOC pathways.

Our current study provides novel cues for previously reported sorghum-insect interactions that may depend on differential VOC profiles. For example, we showed that NOG and BTx623 are differentially susceptible to Asian stem borer, *Ostrinia furnacalis* Guenée.^16^ As BTx623 produced more anisole, it might be working as a repellent to stem borers and/or attract their natural enemies.^61^ *Vice versa*, NOG released more linalool, suggesting that linalool might be actually attracting more herbivores to this cultivar. Further studies are necessary to establish the exact roles of VOCs and HIPVs in sorghum interactions with herbivores, which can be later applied to integrated pest management and plant protection.^62^ The potential impacts of C3 and C4 metabolic networks on VOC emissions also call for further research including multiple species and their comparisons.

## Supplementary Material

Supplemental Material
