# Supplementary figures and images for: Comparative analysis of sorghum (C4) and rice (C3) plant headspace volatiles induced by artificial herbivory
Source: Plant Signal Behav. 2023 Aug 10;18(1):2243064. doi: 10.1080/15592324.2023.2243064 (PMC10730142; doi:10.1080/15592324.2023.2243064)

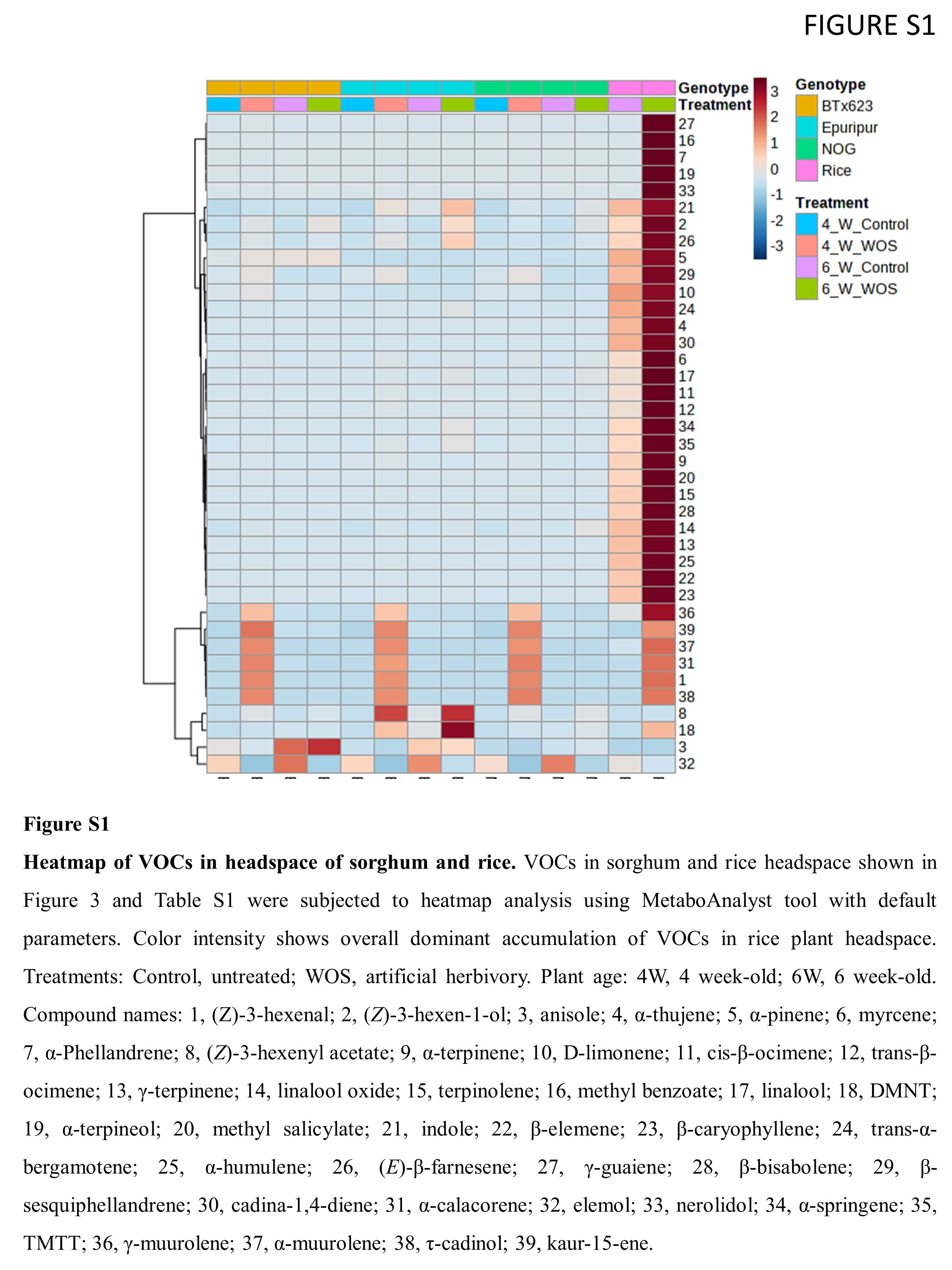

Supplement: Supplemental Material [file KPSB_A_2243064_SM3355.zip › Supplemental Figure S1.JPG]

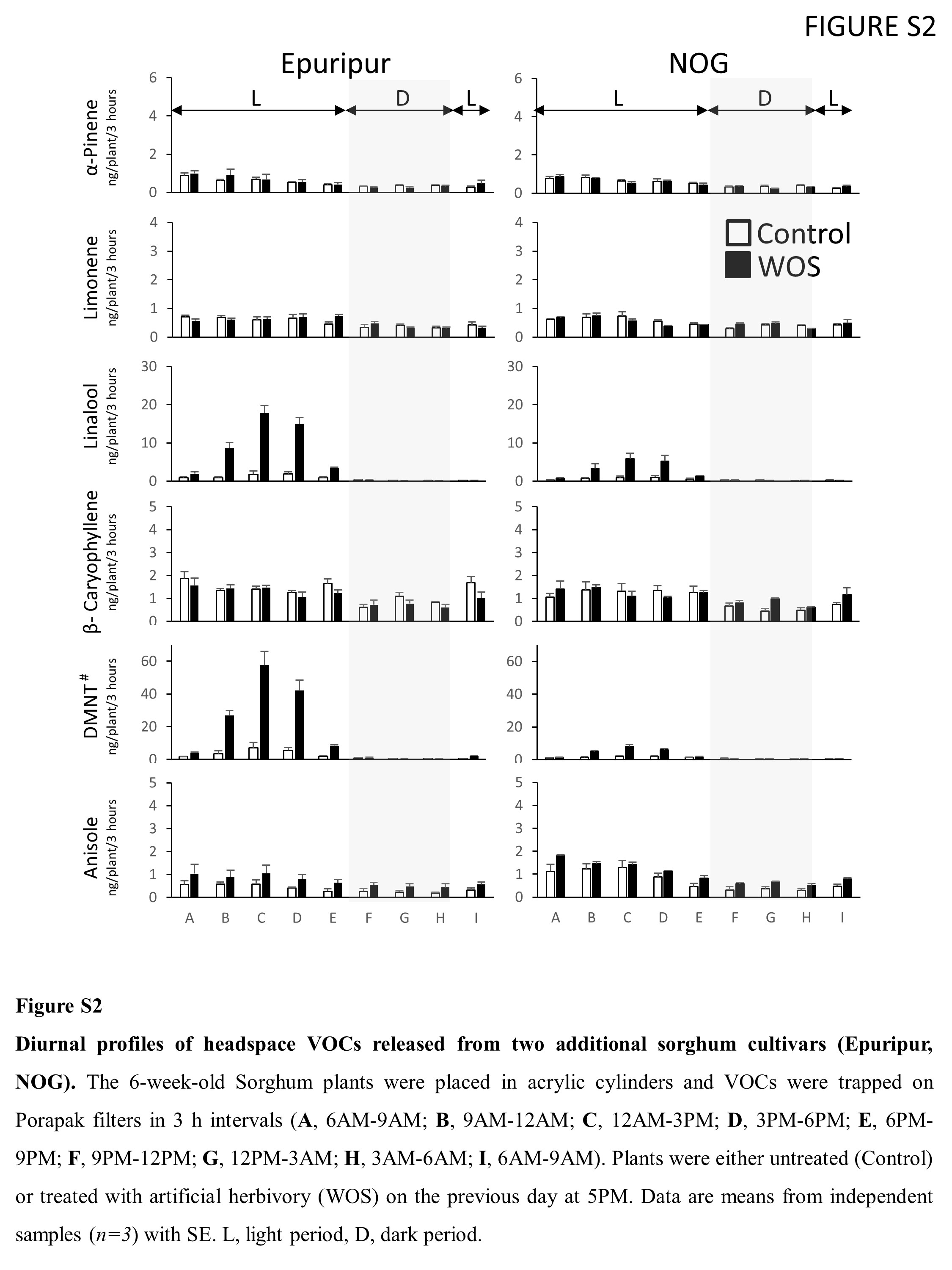

Supplement: Supplemental Material [file KPSB_A_2243064_SM3355.zip › Supplemental Figure S2.JPG]

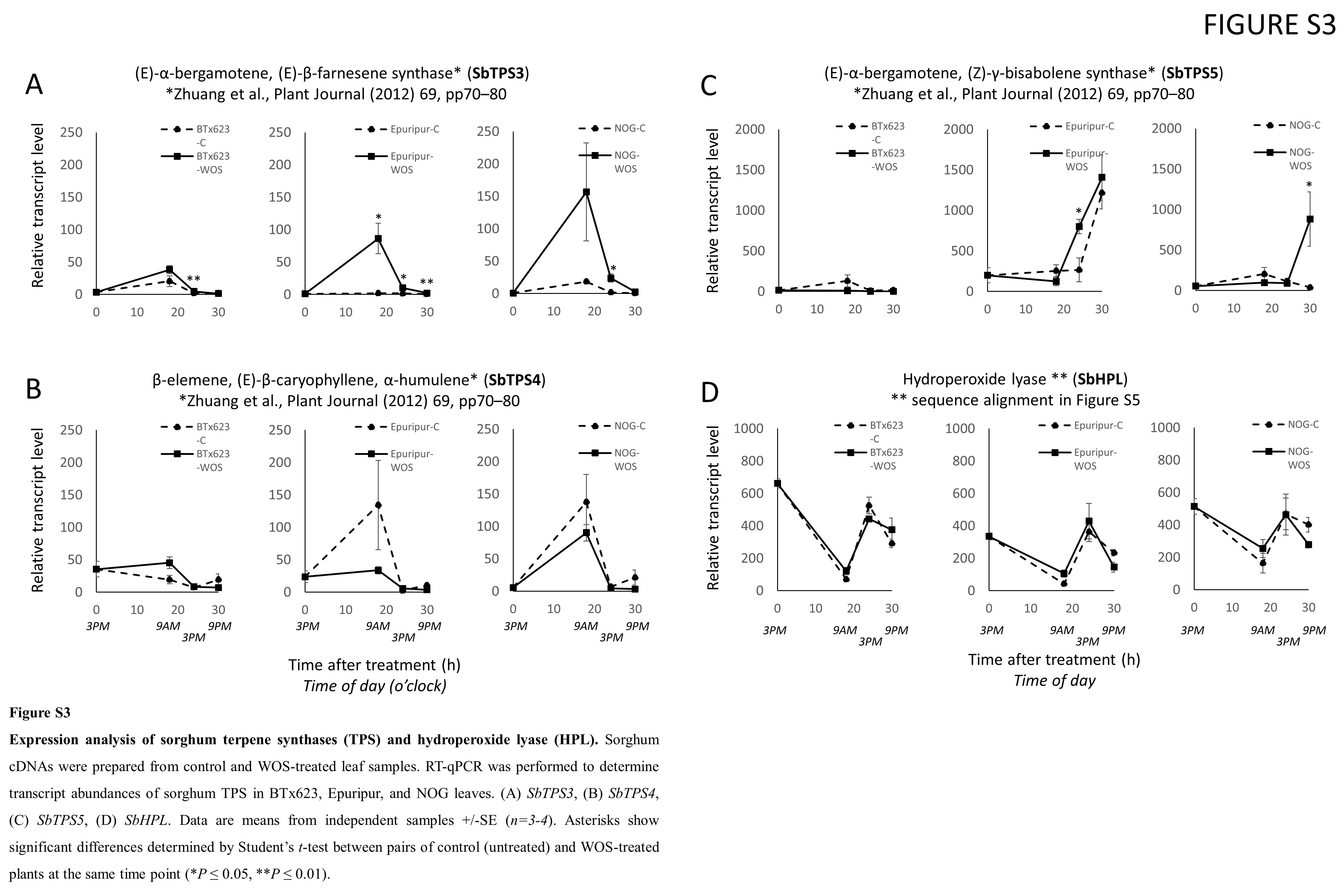

Supplement: Supplemental Material [file KPSB_A_2243064_SM3355.zip › Supplemental Figure S3.JPG]

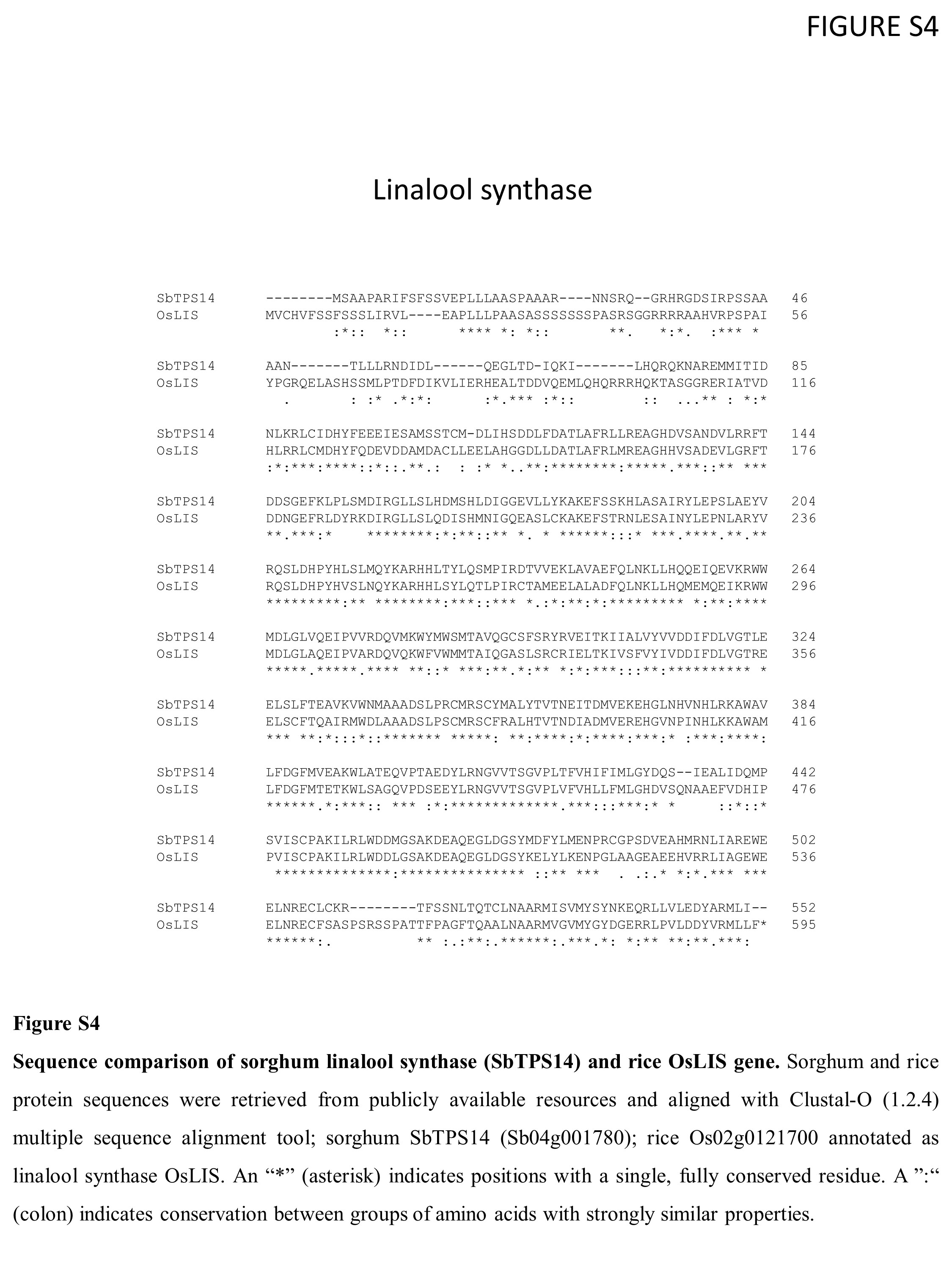

Supplement: Supplemental Material [file KPSB_A_2243064_SM3355.zip › Supplemental Figure S4.JPG]

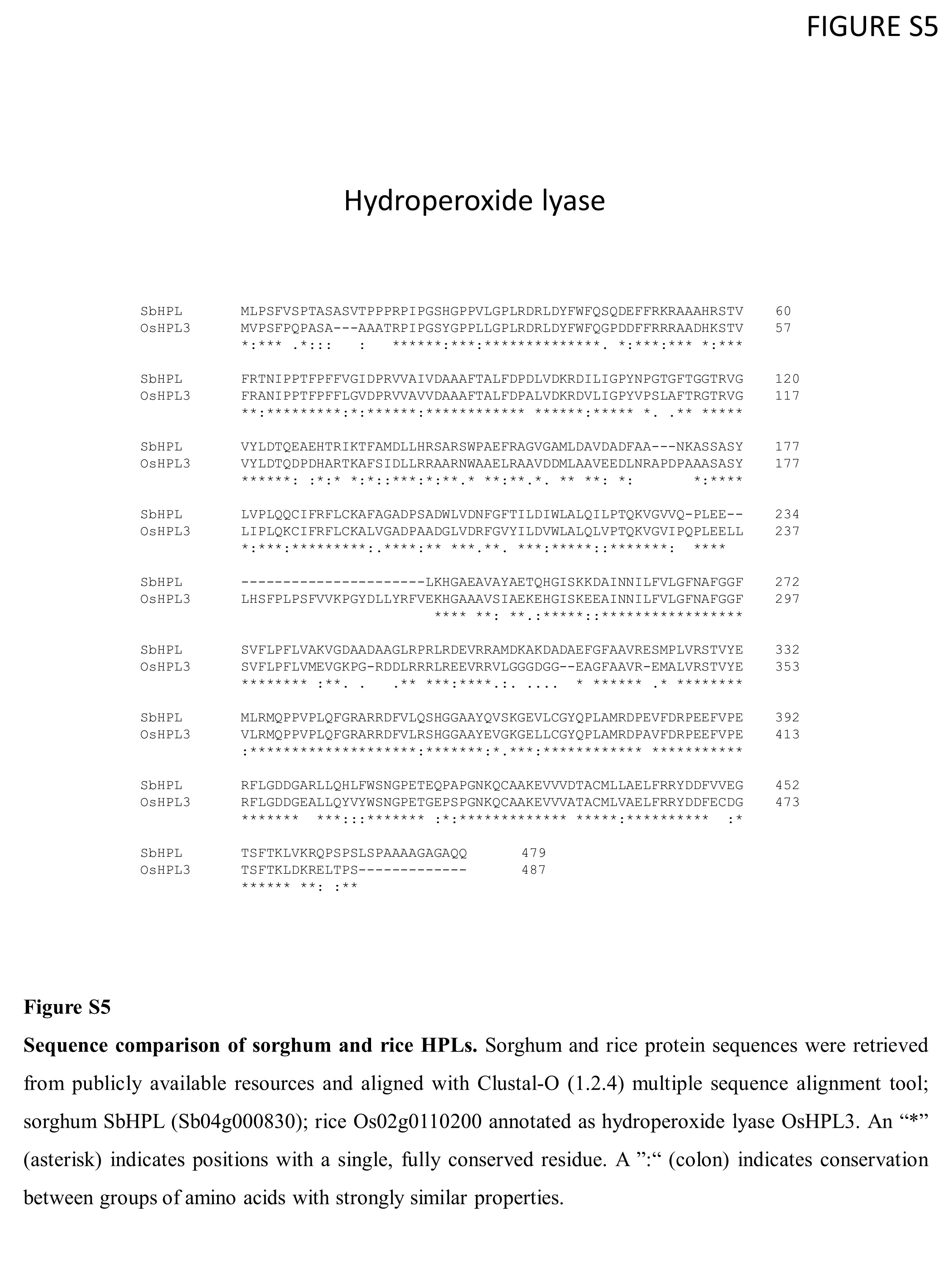

Supplement: Supplemental Material [file KPSB_A_2243064_SM3355.zip › Supplemental Figure S5.JPG]
